# Antioxidant activity of rosemary (*Rosmarinus officinalis* L.) essential oil and its hepatoprotective potential

**DOI:** 10.1186/1472-6882-14-225

**Published:** 2014-07-07

**Authors:** Aleksandar Rašković, Isidora Milanović, Nebojša Pavlović, Tatjana Ćebović, Saša Vukmirović, Momir Mikov

**Affiliations:** 1 Department of Pharmacology, Toxicology and Clinical Pharmacology, Faculty of Medicine, University of Novi Sad, Hajduk Veljkova 3, 21000 Novi Sad, Serbia; 2 High Medical School of Professional Skills, Cara Dušana 254, 11080 Zemun, Serbia; 3 Clinical Center of Vojvodina, Faculty of Medicine, University of Novi Sad, Hajduk Veljkova 3, 21000 Novi Sad, Serbia

**Keywords:** *Rosmarinus officinalis*, Rosemary, Essential oil, Antioxidant enzymes, Hepatoprotection, Oxidative stress

## Abstract

**Background:**

Natural antioxidant products are increasingly being used to treat various pathological liver conditions considering the role of oxidative stress in their pathogenesis. Rosemary essential oil has already being used as a preservative in food industry due to its antioxidant and antimicrobial activities, but it was shown to possess additional health benefits. The aim of our study was to evaluate the protective effect of rosemary essential oil on carbon tetrachloride - induced liver injury in rats and to explore whether its mechanism of action is associated with modulation of hepatic oxidative status.

**Methods:**

Chemical composition of isolated rosemary essential oil was determined by gas chromatography and mass spectrometry. Antioxidant activity was determined *in vitro* using DPPH assay. Activities of enzyme markers of hepatocellular damage in serum and antioxidant enzymes in the liver homogenates were measured using the kinetic spectrophotometric methods.

**Results:**

In this research, we identified 29 chemical compounds of the studied rosemary essential oil, and the main constituents were 1,8-cineole (43.77%), camphor (12.53%), and α-pinene (11.51%). Investigated essential oil was found to exert hepatoprotective effects in the doses of 5 mg/kg and 10 mg/kg by diminishing AST and ALT activities up to 2-fold in serum of rats with carbon tetrachloride - induced acute liver damage. Rosemary essential oil prevented carbon tetrachloride - induced increase of lipid peroxidation in liver homogenates. Furthermore, pre-treatment with studied essential oil during 7 days significantly reversed the activities of antioxidant enzymes catalase, peroxidase, glutathione peroxidase and glutathione reductase in liver homogenates, especially in the dose of 10 mg/kg.

**Conclusions:**

Our results demonstrate that rosemary essential oil, beside exhibiting free radical scavenging activity determined by DPPH assay, mediates its hepatoprotective effects also through activation of physiological defense mechanisms.

## Background

The liver has a central role in the metabolism, transport and clearance of xenobiotics, and is therefore highly susceptible to chemical-induced toxicity. In the Western world, drug‒induced liver injury is a major health care problem and accounts for the majority of acute liver failure cases [1]. It is considered to be idiosyncratic and in addition to genetic factors, environmental and lifestyle factors and pre-existing pathological conditions also determine the susceptibility of individuals to chemical-induced liver injury. The pathophysiological mechanisms of chemical-induced hepatotoxicity are not yet fully understood, but are mostly associated with the metabolic conversion of xenobiotics into reactive oxygen species (ROS), which induce oxidative stress and damage the cellular macromolecules [2]. Oxidative stress, as the imbalance between endogenous generation of ROS and activity of antioxidant systems, has recently been recognized as a key factor in the pathophysiological changes observed in a wide range of liver diseases, such as subclinical hepatitis without jaundice, inflammatory necrotic hepatitis, liver cirrhosis and hepatocellular carcinoma [3,4]. Better understanding the role of oxidative stress in these liver disorders may lead to the appropriate use of antioxidants as a therapeutic approach for liver diseases. Natural antioxidant products, especially phytochemicals, have gained popularity worldwide due to their efficacy and safety. They are increasingly being used to treat various pathological liver conditions and nearly half of the agents used in liver therapy today are either natural products or their derivatives [5].

Rosemary (*Rosmarinus officinalis* L., Lamiaceae) is a woody perennial herb, native to the Mediterranean region, but is now cultivated all over the world as an ornamental and aromatic plant. The leaves of rosemary are commonly used for flavoring foods as a condiment, but this plant has also been widely used for different medicinal purposes. In traditional medicine, rosemary has been used as a stimulant and mild analgesic, and it has been considered as one of the most effective herbs for treating headaches, poor circulation, inflammatory diseases, and physical and mental fatigue. Rosemary has also been used empirically as a choleretic and hepatoprotective agent in folk medicine [6,7]. Most pharmacological effects of rosemary are the consequence of high antioxidant activity of its main chemical constituents, which include carnosol, carnosic acid, ursolic acid, rosmarinic acid, and caffeic acid. The potent antioxidant properties of rosemary have been mainly attributed to its major diterpenes, carnosol and carnosic acid, as well as to the essential oil components [8].

Recently, the essential oils and their active compounds have been of great interest due to their pharmacological properties. Essential oils are complex mixtures of volatile compounds with strong odor that are synthesized in several plant organs and exert diverse ecological functions [9]. Due to their biological activities, the essential oils have been reported to be useful in food preservation, fragrance industry and aromatherapy [10]. Rosemary essential oil (REO) is a colorless or pale yellow liquid, with characteristic odor of the plant, and consists mostly of monoterpenes such as 1,8-cineole, camphor and α-pinene [6]. Due to its antioxidant and antimicrobial activity [11,12], REO is capable to extend the shelf-life of food products and maintain their quality during storage. Therefore, it has already being used as a biopreservative in food industry [13].

In addition to acting as an antioxidant agent, the essential oil isolated from rosemary possesses various health benefits and therapeutic effects. According to the recommendation of European Medicines Agency (EMA) from 2010, REO can be used for treating dyspepsia and mild spasmodic disorders of the gastrointestinal tract, as well as an adjuvant in the relief of minor muscular and articular pain and in minor peripheral circulatory disorders [14]. Besides, the experiments conducted with REO have demonstrated its several notable pharmacological effects, such as anti-inflammatory and antinociceptive [15], antidepressant [16], cognition-enhancing [17], DNA-protective [18] and anticancer effects [19], among others.

The hepatoprotective effects of rosemary have been observed in different experimental models of liver injury. The rosemary methanol extract has been effective against carbon tetrachloride (CCl_4_)-induced acute liver damage [20], and the rosemary water extract could prevent azathioprine-induced acute liver injury in rats [21]. The methanol extract of rosemary could also prevent hepatotoxicity in both prevention and reversion experimental models of liver cirrhosis induced by CCl_4_[22]. To the best of our knowledge, the potential hepatoprotective activity of essential oil from rosemary, which contains monoterpenes as its main active compounds, has not been previously tested *in vivo*. The aim of the present study was to examine the chemical composition of essential oil isolated from the aerial parts of *Rosmarinus officinalis* by GC/MS, to evaluate the protective effect of the obtained essential oil on CCl_4_-induced liver injury in rats and to explore whether its mechanism of action is associated with modulation of hepatic oxidative status.

## Methods

### Plant material and chemicals

Aerial parts of cultivated plants of rosemary were obtained from the Institute for Studies on Medicinal Plants, Dr Josif Pančić, Belgrade, in 2010. A voucher specimen of the plant (*Rosmarinus officinalis* L. 1753 subsp. *officinalis* No 2–1746, det.: Goran Anačkov) was confirmed and deposited in the Herbarium of the Department of Biology and Ecology, Faculty of Sciences, University of Novi Sad. The essential oil, used in our experiments, was isolated from the obtained plant material.

Carbon tetrachloride was obtained from Merck (Darmstadt, Germany). All chemicals for biochemical assays were purchased from Sigma-Aldrich (St Louis, MO, USA), unless otherwise stated.

### Isolation and qualitative and quantitative analysis of essential oil

The essential oil was isolated from the aerial parts of rosemary by hydrodistillation, according to the procedure of the European Pharmacopoeia 4 [23]. n-Hexane was used as a collecting solvent, which was afterward removed under vacuum from the obatined essential oil.

The identification and quantification of chemical constituents of the essential oil were carried out by gas chromatography coupled with flame ionization detection (GC/FID) and mass spectrometric detection (GC/MS). GC/FID analysis was performed using a Hewlett-Packard HP 5890 series II chromatograph equipped with an autosampler and a split/splitless injection system. The capillary column used in this study was HP-5 (25 m × 0.32 mm; film thickness of 0.52 μm), coupled to the flame ionization detector (FID). The injector and detector temperatures were set at 250°C and 300°C, respectively, and the column temperature was programmed from 40 to 260°C at a rate of 4°C/min. The flow rate of hydrogen as a carrier gas was 1 ml/min. A sample of 1% solution of the oil in ethanol (1 μl) was injected in split mode (split ratio, 1:30). GC/MS analysis was carried out using a Hewlett-Packard HP G1800C series II GCD system under the same analytical conditions as in GC/FID. The column HP-5MS (30 m × 0.25 mm; film thickness 0.25 μm) and helium as a carrier gas were used in this analysis. The system was operated in electron ionization (EI) mode at 70 eV, in the mass (m/z) range 40–450 Da.

Identification of essential oil constituents was performed by comparison of obtained mass spectra and retention indexes with those from NIST mass spectra library (IBM-AT, version 2, 1990) and literature data. The quantitative analysis provided the percentage composition of the essential oil components, calculated by FID peak area normalization method.

### Evaluation of *in vitro* antioxidant activity and total phenol content of essential oil

Antioxidant activity of the rosemary essential oil (REO) was evaluated as free radical scavenging capacity (RSC). The ability of REO to donate an electron and scavenge the stable 1,1-diphenyl-2-picrylhydrazyl (DPPH) radical was investigated [24]. The essential oil and methanol solution of α-tocopherol, as a positive control, were mixed with DPPH solution and after 60 minutes, remaining amount of the purple-colored DPPH radical was measured spectrophotometrically at 515 nm. Free radical scavenging capacity was calculated as follows: RSC = 100–100 * A_sample_/A_blank_, where A_blank_ is the absorbance of diluted DPPH solution and A_sample_ is the absorbance of the essential oil/reference. All the assays were carried out in triplicate and the average values were considered. The IC_50_ value, which represents the concentrations of the sample required to cause 50% inhibition of DPPH radical, was estimated by linear regression analysis from the obtained RSC values and was expressed in μl of essential oil per ml.

Phenolic content of REO was assayed using the Folin-Ciocalteu reagent [25]. The essential oil was mixed with Folin-Ciocalteu reagent and sodium carbonate solution in test tube. After being vortexed and incubated in dark for 2 h, absorbance was measured at 740 nm. Estimation of total phenol content was carried out in triplicate and the result was expressed as mg of gallic acid equivalents (GAE) per liter.

### Animals and treatment

The experiment was performed on 7–8 week old albino Wistar rats of both sexes, weighing 200–250 g, obtained from Military Medical Academy, Belgrade. The rats were housed in standard laboratory cages, under a 12 h light–dark cycle, at a constant ambient temperature (23°C) and humidity (30-50%), and were allowed to adapt for 4 weeks before the experiments were started. The animals were maintained on standard pellet diet (LM2, Veterinary Institute, Subotica, Serbia) and allowed access to tap water *ad libitum*. Animal care and all experimental procedures were conducted in accordance with the Guide for the Care and Use of Laboratory Animals edited by Commission of Life Sciences, National Research Council (USA). The study was approved by the Ethical Committee of the University of Novi Sad.

To evaluate the effect of REO on CCl_4_-induced liver injury, the animals were randomly divided into six experimental groups, each containing six individuals, and treated as follows:

• Con S: Control group, saline 1 ml/kg *i.p*.

• Con CCl_4_: saline 1 ml/kg *i.p*. + a single CCl_4_ dose 1 ml/kg *i.p*.

• REO5: rosemary essential oil 5 mg/kg *p.o*.

• REO5 + CCl_4_: rosemary essential oil 5 mg/kg *p.o*. + a single CCl_4_ dose 1 ml/kg *i.p*.

• REO10: rosemary essential oil 10 mg/kg *p.o*.

• REO10 + CCl_4_: rosemary essential oil 10 mg/kg *p.o*. + a single CCl_4_ dose 1 ml/kg *i.p*.

REO was applied by *per os* gavage, previously suspended in saline. The variability in the volume of administered doses was managed by adjusting the concentration to ensure a constant volume (1 ml/kg). REO and saline were administered every day for seven days. On the 7th day, 1 h after the last dose of REO or saline, animals were treated intraperitoneally with CCl_4_ dissolved in olive oil (1:1, 1 ml/kg). After 24 h, rats were anesthetized with urethane (0.75 mg/kg) and sacrificed by cardiopunction, and the samples of blood and liver were taken [26]. The obtained serum was used for determination of standard biochemical parameters. Liver homogenates were prepared from 1 g of liver tissues which were homogenized in a Potter homogenizer with Tris–HCl sucrose buffered solution in a ratio 1:3 (w/v) at 4°C. The parameters of oxidative stress were analyzed in obtained liver homogenates.

### Serum biochemical parameters determination

Total cholesterol and triglycerides levels as biochemical parameters related to lipid status, bilirubin level as a marker of the liver excretory function, activities of enzyme markers of hepatocellular damage, including alanine aminotransferase (ALT) and aspartate aminotransferase (AST), and the concentration of urea, creatinine and uric acid as indicators of renal excretory function were determined in serum. All analyses were performed in triplicate for every sample on the Olympus AU 400 autoanalyzer (Hamburg, Germany) by using commercially available kits based on the well-established spectrophotometric methods, according to the manuals supplied.

Triglycerides and cholesterol were quantitated by enzymatic colorimetric methods. Glycerol phosphate oxidase-peroxidase (GPO-POD) and cholesterol oxidase-peroxidase (CHOD-POD) methods were applied for determination of triglycerides and cholesterol, respectively. Total and direct bilirubin concentrations in serum were estimated by colorimetric DPD method, based on the reaction between bilirubin and 2,5-dichlorophenyldiazonium salt to produce an azobilirubin complex. AST and ALT activities were measured by standard IFCC methods. Serum level of creatinine was measured by modified Jaffe’s method, and determinations of urea and uric acid levels were performed using glutamate dehydrogenase (GLDH-UV) and uricase-peroxidase kinetic methods, respectively.

### Determination of oxidative status in the liver

Oxidative status in the liver was estimated by measuring the levels of lipid peroxidation (LPx) and reduced glutathione (GSH), and the activities of antioxidant enzymes, including catalase (CAT), peroxidase (Px), glutathione peroxidase (GPx), and glutathione reductase (GR) in liver homogenates, using the spectrophotometric methods. All measurements were performed in triplicate for every sample.

The intensity of LPx was estimated by measuring the amount of malondialdehyde, a terminal product of lipid breakdown due to peroxidation damage, using the method of Buege and Aust [27]. The content of GSH was determined by the reaction with 5,5′-dithiobis (2-nitrobenzoic acid), according to the method of Kapetanović and Mieyal [28]. CAT activity was determined by the method based on monitoring the H_2_O_2_ decomposition rate at 240 nm [29], and Px activity by using guaiacol as the enzyme’s substrate [30]. The activities of GPx and GR were determined following the methods of Beutler [31] and Goldberg and Spooner [32], respectively. These tests were based on the measuring the decrease in absorbance caused by the oxidation of NADPH at 340 nm (UV assay).

### Statistical analysis

Data are expressed as mean ± standard error of the mean (SEM). The intergroup variation between various groups was measured by one-way analysis of variance (ANOVA) followed by Tukey’s multiple comparison test. Results were considered statistically significant when p < 0.05. Data were analyzed using SPSS software, version 19 (IBM SPSS, Chicago, IL, USA).

## Results

### REO chemical composition

The essential oil distilled from the rosemary had a pale yellow color and a strong odor, and the obtained yield of the essential oil was 1.03% (v/w in dry matter). The total number of identified chemical constituents was 29, representing 99.87% of the total oil content. The percentage composition of REO is presented in Table 1.

**Table 1:** Chemical composition of the rosemary essential oil

**Compounds**	**RT**-**FID**^**a**^	**RT**-**MS**^**b**^	**RRT**^**c**^	**Percentage content (% m/m)**
Monoterpene hydrocarbons				31.22
Tricyclene	12.640	6.82	0.579	0.23
α-Thujene	12.777	7.00	0.585	0.13
α-Pinene	13.100	7.19	0.600	11.51
Camphene	13.737	7.62	0.629	4.55
Sabinene	14.697	8.47	0.673	0.05
β-Pinene	14.887	8.50	0.681	8.16
β-Myrcene	15.292	9.04	0.700	0.99
α-Phellandrene	15.955	9.43	0.730	0.19
δ^3^-Carene	16.212	9.62	0.742	0.13
α-Terpinene	16.456	9.85	0.753	0.14
p-Cymene	16.782	10.13	0.768	1.23
Limonene	16.970	10.25	0.777	2.80
γ-Terpinene	18.178	11.30	0.832	0.92
α-Terpinolene	19.416	12.31	0.889	0.19
Oxygenated monoterpenes				63.88
1,8-cineole	17.124	10.43	0.784	43.77
Linalool	19.746	12.82	0.904	0.46
Camphor	21.845	14.30	1.000	12.53
Isoborneol	22.318	14.71	1.022	0.53
Borneol	22.655	15.05	1.037	2.97
Terpinen-4-ol	23.059	15.44	1.056	0.56
α-Terpineol	23.548	15.93	1.078	1.53
γ-Terpineol	23.802	16.17	1.090	0.40
Bornyl acetate	27.185	19.14	1.244	1.13
Sesquiterpene hydrocarbons				4.77
α-Copaene	30.598	22.03	1.401	0.12
Longifolene	31.866	22.93	1.459	0.18
β-Caryophyllene	32.240	23.41	1.476	3.93
α-Humulene	33.406	24.45	1.529	0.36
Germacrene D	34.029	25.18	1.558	0.08
δ-Cadinene	35.528	26.59	1.626	0.10
Total identified				99.87
Number of compunds identified				29

^a^RT-FID - retention time in GC/FID system; ^b^RT-MS - retention time in GC/MS system; ^c^RRT - relative retention time with respect to camphor.

The essential oil contains a complex mixture of 95.10% of monoterpenes and 4.77% of sesquiterpenes. It was found to be composed mainly of oxygenated monoterpenes (63.88%), followed by monoterpene hydrocarbons (31.22%) and sesquiterpene hydrocarbons (4.77%). The major compounds that were identified and quantitated by GC-FID and GC-MS were 1,8-cineole (43.77%), camphor (12.53%), α-pinene (11.51%), β-pinene (8.16%), camphene (4.55%), and β-caryophyllene (3.93%) (Additional files 1 and 2).

### *In vitro* antioxidant activity of REO

The antioxidant activity of REO was evaluated by the DPPH free radical scavenging test and the result was presented by IC_50_ value, defined as the concentration of the antioxidant needed to scavenge 50% of DPPH present in the test solution. The results demonstrated that the essential oil had a strong radical scavenging activity with an IC_50_ value of 77.6 μl/ml. A comparison was made with α-tocopherol (vitamin E), well-known potent antioxidant and free radical scavenger, which exhibited an IC_50_ value of 25.3 μg/ml. According to that, 1 μg of α-tocopherol was found to be equivalent to 3.1 μl of the studied REO.

Antioxidant activity of rosemary essential oils was reported to be derived partly from the presence of phenolic groups [33]. We measured total phenol content in order to determine if this class of chemical compounds contributes to free radical scavenging capacity of REO, besides monoterpenes. Total phenol content of investigated REO was measured using the Folin-Ciocalteu method and the relatively small amount of total phenols of 153.35 mg GAE/l was determined.

### Effects of REO on CCl_4_-induced serum biochemical parameters

In our study, we investigated the concentrations of triglycerides and cholesterol in serum in order to evaluate the effect of REO on metabolic function of the liver. CCl_4_ induced a slight decrease in triglycerides and cholesterol levels which was partially recovered after administration of REO in a dose of 5 mg/kg. The intake of REO alone did not affect significantly the metabolic function of the liver (Table 2).

**Table 2:** Effects of the rosemary essential oil on biochemical parameters in serum

	**Con S**	**Con CCl**_**4**_	**REO5**	**REO5 + CCl**_**4**_	**REO10**	**REO10 + CCl**_**4**_
Triglycerides (mmol/l)	0.81 ± 0.08	0.52 ± 0.04	0.79 ± 0.06	0.63 ± 0.09	1.03 ± 0.10^#^	0.42 ± 0.03^*^
Cholesterol (mmol/l)	1.56 ± 0.08	1.38 ± 0.08	1.39 ± 0.03	1.54 ± 0.06	1.68 ± 0.06	1.31 ± 0.13
Total bilirubin (μmol/l)	2.05 ± 0.15	4.02 ± 0.35^*^	2.24 ± 0.06^#^	3.94 ± 0.35^*^	2.55 ± 0.07^#^	6.22 ± 0.50^*,#^
Direct bilirubin (μmol/l)	0.35 ± 0.03	1.32 ± 0.24^*^	0.38 ± 0.05^#^	1.16 ± 0.15^*^	0.55 ± 0.02^#^	2.81 ± 0.29^*,#^
AST (U/l)	125.3 ± 4.2	3185.0 ± 477.9^*^	130.2 ± 6.2^#^	2701.7 ± 583.0^*^	143.7 ± 7.9^#^	1648.6 ± 104.4^*,#^
ALT (U/l)	49.7 ± 1.7	966.8 ± 244.4^*^	63.8 ± 4.3^#^	595.4 ± 143.7	65.7 ± 2.1^#^	893.5 ± 200.8^*^
Urea (mmol/l)	7.28 ± 0.25	7.72 ± 0.17	7.65 ± 0.08	7.98 ± 0.29	7.45 ± 0.24	6.33 ± 0.29^#^
Creatinine (μmol/l)	46.0 ± 1.18	53.2 ± 0.70^*^	44.2 ± 1.14^#^	51.5 ± 1.59^*^	50.3 ± 0.42	47.7 ± 0.99^#^
Uric acid (μmol/l)	62.0 ± 1.73	104.0 ± 12.02^*^	53.0 ± 2.86^#^	74.3 ± 5.86^#^	60.7 ± 3.69^#^	79.2 ± 6.65

All values are expressed as mean ± SEM. (*) significantly different from Con S group; (^#^) significantly different from Con CCl_4_ group; p < 0.05.

The treatment of animals with CCl_4_ induced statisticaly significant increase in both total and direct bilirubin levels in serum. Increased concentration of bilirubin in serum indicate impaired excretory function of the liver. This effect was additionally exacerbated after the co-treatment with REO in the dose of 10 mg/kg. To confirm that CCl_4_-induced liver injury had occurred, AST and ALT activities were measured in serum. AST and ALT activities were increased 25- and 19-fold respectively in Con CCl_4_ group compared to saline-treated group, and this is known to occurs mainly due to leakage of these enzymes from damaged hepatocytes into the bloodstream. The treatment of animals with REO attenuated these parameters of hepatotoxicity, especially in the group treated with REO in a dose of 10 mg/kg in which AST activity was significantly decreased compared to CCl_4_-treated group.

Serum concentrations of urea, creatinine and uric acid, as biomarkers of renal function, were also examined. These biochemical parameters were increased in CCl_4_-treated group, indicating its nephrotoxicity, but were significantly restored after REO administration.

### Effects of REO on CCl_4_-induced oxidative stress

In order to analyze the antioxidative potential of REO to prevent the CCl_4_-induced biochemical changes in the liver of rats, we measured the malondialdehyde (MDA) and glutathione (GSH) levels and activities of catalase (CAT), peroxidase (Px), glutathione peroxidase (GPx), and glutathione reductase (GR) as biomarkers of REO effects.

**Figure 1:**
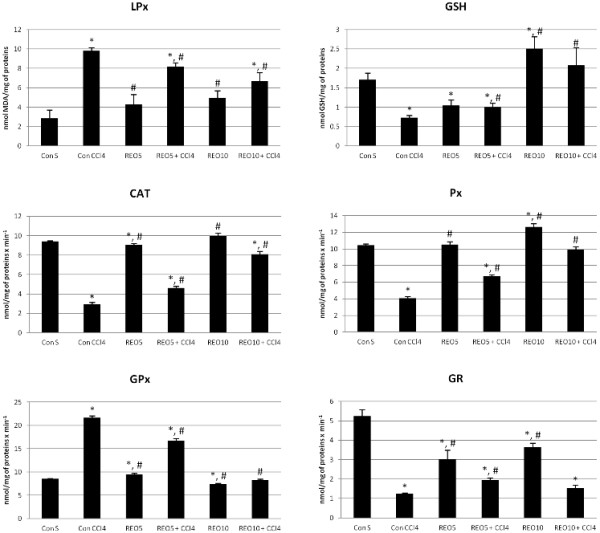
**Levels of lipid peroxidation (LPx), glutathione (GSH), catalase (CAT), peroxidase (Px), glutathione peroxidase (GPx), and glutathione reductase (GR) in liver homogenates.** (*) significantly different from Con S group; (^#^) significantly different from Con CCl_4_ group; p < 0.05.

The single dose of CCl_4_ induced 3.5-fold increase in MDA level compared to the negative control group of rats 24 h after the administration, suggesting the oxidative damage of cell membranes. This was accompanied by the significant reduction of GSH concentration (0.73 ± 0.05 nmol vs. 1.71 ± 0.17 nmol GSH/mg of proteins in control group), and activities of all examined antioxidant enzymes, except GPx, showing increased activity in the liver homogenates (Figure 1).

Our results showed that daily administration of REO could partially normalize altered biochemical parameters in the experimental model of acute liver injury. Pre-treatment with REO in both doses during 7 days significantly reversed the oxidative stress-related biochemical parameters in liver homogenates, especially in the dose of 10 mg/kg in which REO reversed these parameters near to the values of negative control group. Besides, administration of REO alone also modified significantly most of the studied oxidative stress biomarkers and improved oxidative status of the liver.

## Discussion

In this research, we determined the chemical composition of the studied REO, its free radical scavenging activity, and its potential to ameliorate liver injury induced by pro-oxidant agent CCl_4_. Earlier studies regarding the chemical composition of essential oils isolated from rosemary indicate the existence of three main chemotypes. The main component of the Tunisian, Turkish, Moroccan and Italian oils is 1,8-cineole with usually over 40%, whereas most Spanish, French and Greek oils have 1,8-cineole, α-pinene and camphor with approximately equal ratios (20-30%). The myrcene-rich rosemary oil chemotype has been reported in South America [13]. The main constituents in the rosemary essential oil investigated in our study were 1,8-cineole (43.77%), camphor (12.53%), and α-pinene (11.51%). Therefore, REO from our study can be categorized in the Morocco/Tunisian type. It should be noted that significant variations in the chemical composition of rosemary essential oils have been reported. Several factors were found to influence the composition of essential oils, including the geographic origin, part of the plant, season of harvesting, hence the phenological stage of the plant, and also the essential oil isolation method [9]. All effects of REO should be therefore carefully examined, considering the chemical composition of the investigated oil.

We evaluated *in vitro* antioxidant activity of REO and demonstrated a relatively high DPPH radical scavenging capacity with IC_50_ value of 77.6 μl/ml. Our results are in agreement with previous data reported on the antioxidant activity of essential oils of rosemary. Free radical scavenging activity, and chemical composition of the oil we investigated as well, were similar to the essential oil obtained from the rosemary cultivated in South-Western Tunisia, as demonstrated in the study of Kadri et al. [10]. IC_50_ values generally vary considerably among studies, which can be explained by different chemical compositions of rosemary essential oils. The chemotype of rosemary myrcene-rich essential oils has been recently shown to have the highest antioxidant activity [13]. In the study aimed to evaluate and compare the free radical scavenging properties of REO with three of its components (1,8-cineole, α-pinene and β-pinene), REO exhibited lower IC_50_ value and hence greater antioxidant activity than all its components, and than the synthetic antioxidant butylated hydroxytoluene (BHT) as well [34]. This means that in addition to the major compounds, minor components may also contribute significantly to the activity of REO. In another study, the essential oils from five plants that are widely used in a Mediterranean diet (oregano, thyme, clove, sage, and rosemary) were tested for their antioxidant properties, using six *in vitro* methods, and the results showed that REO had the highest iron-chelating ability. Besides, REO exhibited total phenol content of 225 mg GAE/l [33], which can be considered as a low source of phenols in comparison to other investigated essential oils, and is relatively similar to the results of our study. This means that oxygenated monoterpenes, probably monoterpenoid ketones with established antioxidant properties, may have the greatest contribution to the antioxidant capacity of REO.

The hepatoprotective effects of REO were investigated by measuring ALT and AST activities, as enzyme markers of hepatocellular damage, in serum. In our study, we also evaluated the effects of REO on metabolic and excretory functions of the liver, and renal excretory function as well. It is well known that CCl_4_, as a highly toxic and pro-oxidant agent, produce the damage of hepatocytes to cause large increases in ALT and AST activities and elevation in bilirubin content in serum. REO was found to exert hepatoprotective effects in both doses by diminishing AST and ALT activities in serum, as the most specific markers of liver injury, compared to CCl_4_-treated group. Similarly, results of the study by Sotelo-Félix et al. [20] and Gutiérrez et al. [22] showed that methanolic extract of rosemary in a dose of 200 mg/kg could restore the elevated ALT activity in both acute liver damage and liver cirrhosis experimental models. Interestingly, co-administration of REO in the dose of 10 mg/kg with CCl_4_ resulted in exacerbation of bilirubin values in serum indicating impaired excretory function of the liver. On the contrary, REO in the dose of 5 mg/kg did not exert that effect. This is in agreement with previous findings that many monoterpenes do not exhibit dose-dependent effects and that it is necessary to find the most appropriate dose range that shows effectiveness [35]. Among numerous terpenoids, camphor, a bicyclic monoterpene which is present in our REO in a relatively high amount (12.53%), is a potentially toxic compound and a small amount can cause poisoning in children. Camphor is neurotoxic, and is also toxic to the liver, but the mechanisms are still to be elucidated. It was demonstrated that camphor is oxidized to 5-exo-hydroxyfenchone derivatives in human liver microsomes, and that might contribute to hepatic excretory impairment after administration of REO in the dose of 10 mg/kg [36,37]. Monoterpene α-pinene might also exert toxic effects in high doses. It was demonstrated that α-pinene exhibited cytotoxicity on cultured human blood cells when applied in high concentration (200 mg/l) [38], and this compound was also confirmed to be porphyrogenic and therefore especially hazardous to those with defects in hepatic heme synthesis [39].

In addition to hepatoprotective effects, as indicated by AST and ALT values, the nephroprotective potential of REO was also observed in our study. The intake of REO in a dose of 10 mg/kg produced statisticaly significant diminution in urea and creatinine levels compared to Con CCl_4_ group. These results suggest the ability of REO to restore impaired renal excretory function. This is in accordance with results of the study by Sakr and Lamfon [40], indicating that water extract of rosemary could reduce the levels of urea and creatinine in serum of rats, when compared to CCl_4_-treated group of animals.

Considering the role of reactive oxygen species (ROS) in chemical-induced liver injury and various liver diseases, we used the model of acute liver damage induced by CCl_4_ since the oxidative stress is the main mechanism involved in hepatotoxicity of CCl_4_. This compound is metabolized via reductive dehalogenation involving cytochrome P450, which is accompanied by formation of the radicals CCl_3_^•^ and Cl_3_COO^•^ causing the peroxidation of membrane lipids. MDA is one of the well-known secondary products of lipid peroxidation, and it was used as an indicator of cell membrane injury. As demonstrated in Figure 1, REO showed ability to prevent CCl_4_-induced increase in MDA level, which suggests that REO can preserve cellular integrity and this may be the consequence of free radical scavenging activity determined by DPPH assay. Previous findings showed that methanolic extracts of rosemary, although different in chemical composition from REO, could also inhibit lipid peroxidation in the liver of CCl_4_-treated rats [20]. In addition to antilipoperoxidant activity, REO was also found to efficiently reduce the levels of hydrogen peroxide (H_2_O_2_)- and 2,3-dimethoxy-1,4-naphthoquinone (DMNQ)-induced oxidative damage of DNA in isolated rat hepatocytes, and testicular cells as well [18,41].

The effects of ROS are balanced by antioxidant defense system involving a variety of enzymatic and non-enzymatic mechanisms. GSH is the main non-enzymatic endogenous antioxidant in the cells. Antioxidant enzymes Px, GPx and CAT catalyze the reduction of peroxides to alcohols or water. GR reduces glutathione disulfide (GSSG), generated during the reduction of peroxides, to the sulfhydryl form of glutathione (GSH) [4]. In our study, we investigated also the effects of REO on antioxidant defense system of hepatocytes, and our results showed that administration of CCl_4_ induced the significant depletion of GSH level in liver homogenates. In addition to serving as a cofactor for several antioxidant enzymes, GSH can interact directly with certain ROS to scavenge them, and therefore is used in detoxifying CCl_4_ metabolites. Depletion of GSH was accompanied by a significant reduction of CAT, Px and GR activities, which can be explained by their inactivation induced by excessive ROS production. Interestingly, activity of the antioxidant enzyme GPx was increased in the liver after administration of CCl_4_. Similar results were obtained in the study of Hsiao et al. [42], where it was demonstrated that CCl_4_ induced increase of GPx activity and reduction of CAT activity. These enzymes are activated in the defense against oxidative cell injury. CAT has an important role in the elimination of ROS derived from the redox process of xenobiotics in the liver, and it was suggested that CAT is easily inactivated by lipid peroxides [43]. On the other hand, under oxidative stress conditions, GPx can be upregulated as the adaptive response of the hepatocytes to oxidative damage, and GSH is then largely consumed, which is in accordance with results of our study. The essential oil isolated from rosemary significantly reversed all these biochemical parameters referring to oxidative status of the liver, especially in the dose of 10 mg/kg.

Hepatoprotective activity of investigated essential oil isolated from rosemary can be attributed to 1,8-cineole, as its major compound. This monoterpene was reported to have various pharmacological effects, such as smooth muscle relaxant, anti-inflammatory, antioxidant and hypotensive. In an *in vivo* murine model of septic shock syndrome induced by D-galactosamine/lipopolysaccharide that is characterized by early apoptosis and subsequent lysis of hepatocytes, 1,8-cineole not only suppressed increase in liver weight and the elevation in serum transaminase activity, but also prevented the necrosis and haemorrhage to a greater extent than dexamethasone. Hepatoprotection was suggested to be associated with a reduction in TNF-α serum concentration [44]. In rats treated with the environmental contaminant 2,3,7,8-tetracholorodibenzo-p-dioxin (TCDD) for 30 days, activities of antioxidant enzymes GPx and CAT, and GSH levels in liver were significantly decreased, whereas MDA levels were significantly increased compared to the non-treated group. When given together with TCDD, 1,8-cineole increased GPx, CAT, GSH levels, while reducing MDA concentration close to the level of the control group, suggesting activation of antioxidant defense systems as one of the mechanisms of hepatoprotection induced by 1,8-cineole [45]. Similarly, it was found that 1,8-cineole treatment improved colonic oxidative balance in rats with TNBS-induced colitis, since it was able to reduce the myeloperoxidase activity, a marker of polymorphonuclear leukocyte accumulation and could restore the tissue levels of GSH as the major intracellular antioxidant [46]. Besides 1,8-cineole, antioxidant activity was determined also for α-pinene, a bicyclic monoterpene that is present in the studied essential oil in our study in a relatively high amount (11.51%), and this compound could contribute to hepatoprotective activity of REO as well [38]. The contribution of various compounds that are present in small amounts in the essential oil could be also important for the effects on liver, and their mechanisms of action are still to be elucidated.

## Conclusions

In summary, the present results demonstrate that administration of REO, exhibiting free radical scavenging activity determined by DPPH assay, exerts beneficial effects on preventing CCl_4_-induced hepatotoxicity in rats by limiting the extent of lipid peroxidation and hence cell membranes injuries. Considering the significant impact on activities of examined antioxidant enzymes, it is clear that REO mediates its hepatoprotective effects not only through scavenging of harmful free radicals, but also through activation of physiological defense mechanisms. It should be emphasized that there have been considerable variations in the chemical composition of essential oils obtained from rosemary, and for this reason, the use of REO in preventing and/or treatment of various liver diseases requires the identification of active ingredients and further investigations on their mechanisms of action.

## Competing interests

The authors declare that they have no competing interests.

## Authors’ contributions

Experimental work was done by IM under the supervision of AR who designed all the assays. TĆ performed the antioxidant enzymes analyses. SV carried out a part of the experiments. NP analyzed the data and wrote the manuscript. MM was coordinator of the project. All authors read and approved the final manuscript.

## Pre-publication history

The pre-publication history for this paper can be accessed here:

http://www.biomedcentral.com/1472-6882/14/225/prepub

## Supplementary Material

Additional file 1 Normalized gas chromatogram of the rosmary essential oil giving relative retention times (RRT) with respect to camphor and relative amounts of 29 components. Click here for file

Additional file 2 Mass spectrum of the rosmary essential oil. Click here for file
