# Supplementary figures and images for: Antioxidant activity of rosemary (Rosmarinus officinalis L.) essential oil and its hepatoprotective potential
Source: BMC Complement Altern Med. 2014 Jul 7;14:225. doi: 10.1186/1472-6882-14-225 (PMC4227022; doi:10.1186/1472-6882-14-225)

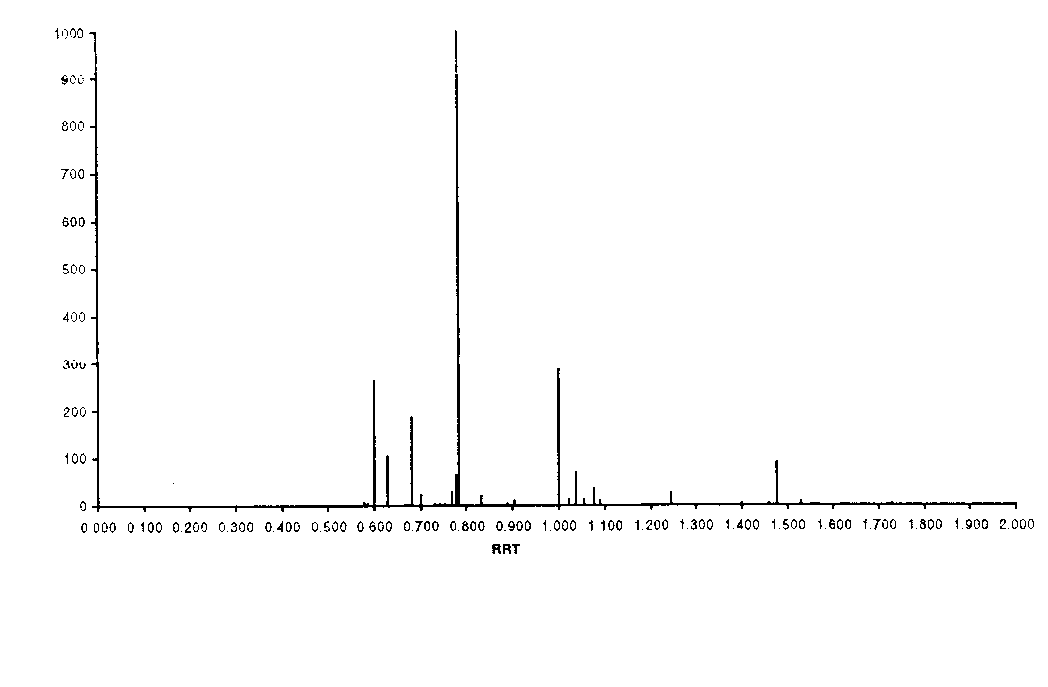

Supplement: Additional file 1 — Normalized gas chromatogram of the rosmary essential oil giving relative retention times (RRT) with respect to camphor and relative amounts of 29 components. [file 1472-6882-14-225-S1.zip › 2068215165115223_add1.bmp]

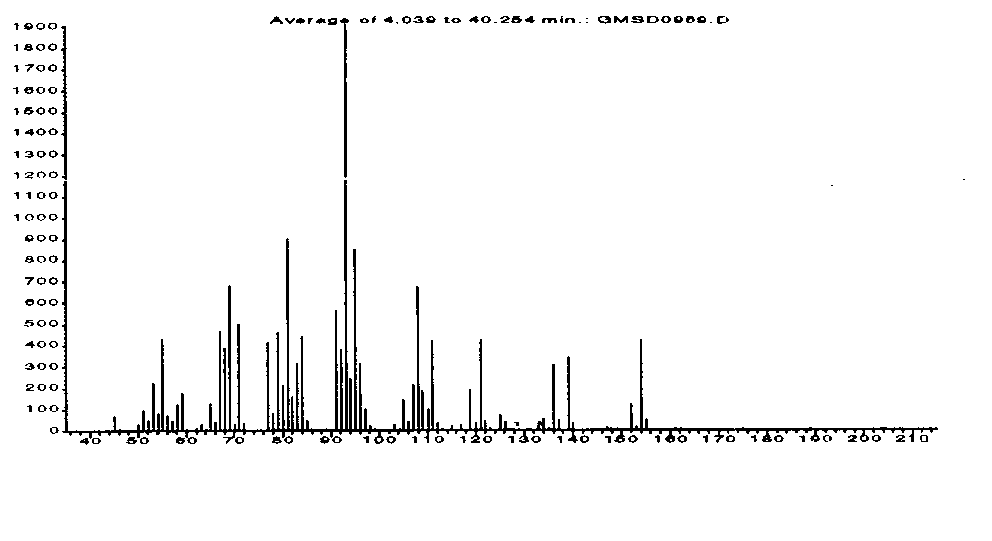

Supplement: Additional file 2 — Mass spectrum of the rosmary essential oil. [file 1472-6882-14-225-S2.zip › 2068215165115223_add2.bmp]
